# The Chemokine CCL3 Promotes Experimental Liver Fibrosis in Mice

**DOI:** 10.1371/journal.pone.0066106

**Published:** 2013-06-17

**Authors:** Daniel Heinrichs, Marie-Luise Berres, Andreas Nellen, Petra Fischer, David Scholten, Christian Trautwein, Hermann E. Wasmuth, Hacer Sahin

**Affiliations:** Medical Department III, University Hospital Aachen, Aachen, Germany; French National Centre for Scientific Research, France

## Abstract

Liver fibrosis is associated with infiltrating immune cells and activation of hepatic stellate cells. We here aimed to investigate the effects of the CC chemokine CCL3, also known as macrophage inflammatory protein-1α, in two different fibrosis models. To this end, we treated mice either with carbon tetrachloride or with a methionine- and choline-deficient diet to induce fibrosis in *CCL3* deficient and wild-type mice. The results show that the protein expression of CCL3 is increased in wild-type mice after chronic liver injury. Deletion of *CCL3* exhibited reduced liver fibrosis compared to their wild-type counterparts. We could validate these results by treating the two mouse groups with either carbon tetrachloride or by feeding a methionine- and choline-deficient diet. In these models, lack of *CCL3* is functionally associated with reduced stellate cell activation and liver immune cell infiltration. *In vitro*, we show that CCL3 leads to increased proliferation and migration of hepatic stellate cells. In conclusion, our results define the chemokine CCL3 as a mediator of experimental liver fibrosis. Thus, therapeutic modulation of CCL3 might be a promising target for chronic liver diseases.

## Introduction

Liver fibrosis frequently progresses to end-stage liver disease including liver cirrhosis and hepatocellular carcinoma, which are mainly caused by viruses, metabolic diseases and alcohol [1], [2]. It is a result of a chronic wound healing process leading to an inflammatory and fibrotic response [3]. Hepatic stellate cells (HSCs), the main fibrogenic resident cells in the liver, play an essential role during this fibrotic response. Following a fibrogenic stimulus, hepatic stellate cells transdifferentiate from quiescent vitamin A-storing cells into activated cell types which produce a large amount of ECM proteins. The inflammatory response during chronic hepatocyte injury triggers the accumulation of immune cells in the liver consisting of e.g. T cells, macrophages, and dendritic cells [4]. The recruitment of these cells is orchestrated by the pattern of cytokines and chemokines which are secreted by activated liver resident cells [5], [6]. Among these cells, hepatic stellate cells secrete a wide range of chemokines (CCL2, CCL3, CCL5 CCL11, CXCL8, CXCL9, and CXCL10), thereby influencing the quantity and also the quality of the inflammatory response [7], [8]. Beside regulation of liver inflammation, the chemokines CCL2, CCL5 and CXCL9 has also been identified to directly influence the biology of hepatic stellate cells [8]–[10].

In the present study, we focus on the chemokine CCL3, which is also known as macrophage inflammatory protein-1α. Murine CCL3 is located on chromosome 11 and is encoded by a single-copy gene [11]. It consists of three exons and two introns, which codes for a pre-protein of 92 amino acids [12]. CCL3 is a 7.8 kDa CC chemokine which is strongly expressed by numerous cell types including T cells, macrophages, neutrophils, endothelial and also stellate cells upon injury [13]–[15].

This chemokine recruits several cell types, like T cells, neutrophils and eosinophils [16]–[18] to site of inflammation by interacting with its receptors CCR1 and CCR5 [19]. Interestingly, hepatic stellate cells also express CCR5, identifying these cells as a source as well as a target of CCL3 within the liver. Moreover, *CCR1* and *CCR5* deficient mice showed a lower degree of liver fibrosis after chronic carbon tetrachloride (CCl_4_) treatment or bile duct ligation (BDL) [13]. Studies of fibrogenesis in *CCR1* and *CCR5* chimeric mice revealed a divergent function of these chemokine receptors in the liver. CCR1 mediates its pro-fibrotic effects predominantly through hematopoietic cells, whereas CCR5 mediates liver fibrosis mainly through resident liver cells [13]. CCL5 seems to be a crucial mediator of this chemokine pathway as *CCL5* ^−/−^ mice also show reduced liver fibrosis induced by carbon tetrachloride (CCl_4_) or methionine- and choline-deficient (MCD) diet. Importantly, administration of mice with Met-CCL5, a CCR1/CCR5 antagonist, attenuated liver fibrosis and accelerated the regression of fibrosis during follow-up [10]. A crucial role for this chemokine has also been established in Concanavalin A (ConA) mediated liver injury [20], a model of T cell mediated hepatitis [21]. In this model, *CCL3^−/−^* mice were significantly rescued from liver failure due to reduced recruitment of CCR1-expressing CD4^+^ T cells [20]. The effects of CCL3 make this CCR1/CCR5 ligand attractive for further evaluation in experimental liver fibrosis.

In line with these previous data, we here show that CCL3 is an important mediator of experimental liver fibrosis by its dual effects on hepatic stellate cells and intrahepatic immune environment. Thus, CCL3 represent a potential target for antifibrotic therapies.

## Materials and Methods

### Murine *in vivo* Experiments

Male C57BL/6 wild-type (WT, *CCL3^+/+^,* 8–10 weeks) mice were routinely purchased from the Jackson Laboratory and acclimated for at least 1 week before any experiments were conducted. *CCL3* deficient (*CCL3^−/−^*) mice, also purchased from the Jackson Laboratory, were maintained on C57BL/6 background and bred in our animal housing facility. Only male littermates (8–10 weeks) were used in experiments. All *in vivo* experiments were performed following approval by the state animal protection board at the Bezirksregierung Cologne, Germany (Permit Number: 8.84–02.04.20.11). *CCL3^−/−^* and wild-type mice were subjected to two different fibrosis models. In the first experiment, *CCL3^−/−^* and WT mice received intraperitoneal injections of carbon tetrachloride (CCl_4_; 0.6 ml/kg bodyweight) for six weeks. Three days after the last injection, the mice were sacrificed [10]. In the second experiment, the mice were fed a methionine- and choline-deficient diet (MCD; MP Biomedicals Europe) for eight weeks. In both models the size were n = 8 per group.

### Quantification of Liver Fibrosis

In all animals, liver fibrosis was determined by quantitative analysis of the Sirius red staining from liver sections. Liver sections were quantified by the Sirius red-positive area using the NIH ImageJ software (http://rsbweb.nih.gov/). Moreover, the collagen-specific amino acid hydroxyproline were determined in the liver samples. The hydroxyproline contents were measured colorimetric as described previously [22].

### Quantification of Hepatocellular Damage

The terminal deoxynucleotidyl transferase (TdT)-mediated dUTP nick-end labelling (TUNEL) assay (Roche) was performed to detect and quantify hepatocellular damage within the liver. The percentage of TUNEL^+^ cells was determined in 3 independent magnification fields per mouse.

### Expression Analysis of Fibrosis and Inflammation Related Genes

Isolation of total RNA from snap-frozen liver samples were performed and total RNA was transcribed into cDNA with the RevertAid™ Premium First Strand cDNA Synthesis Kit (Fermentas). Following quantitative RT-PCR for the genes, *TGF-β1*, *Col1a1*, *TIMP1*, *MMP9*, *α-SMA*, *SREBP1*, *Fas*, *TNF-α*, *IL-1β*, *MCP-1* and *IL-2* was executed with Assay-on-Demand (Applied Biosystems).

### CCL3 ELISA

For protein isolation, liver samples were homogenized in RIPA buffer with protease inhibitor (Mini Complete Protease Inhibitor Cocktail Tablets, Roche Applied Science) and centrifuged; supernatants were collected and stored at −80°C. After thawing, the hepatic CCL3 contents were measured in supernatants using a murine ELISA (R&D Systems) in duplicate, following the manufacturer’s instructions. The OD of the samples was determined at a wavelength of 450 nm. CCL3 concentrations are expressed in pg per mg of total liver protein.

### Hepatic Immune Cell Isolation and Flow Cytometry Analysis

Flow cytometry analysis of hepatic immune cells was performed by isolating single cell suspension from freshly harvested liver [23]. The cells were isolated by mechanical and enzymatic digestion. Viable white blood cells were separated by centrifugation for 20 minutes at 800 g. After the density gradient centrifugation, the isolated peripheral blood mononuclear cells (PBMCs) were washed with Hank’s buffer supplemented with 1% BSA and 2 mM EDTA. Cells were stained with antibodies for CD45, CD3, CD4, CD8 and NK1.1 (eBioscience) and then measured with BD FACSCanto II (BD Bioscience).

### Immunohistochemical Staining of Murine Liver Tissue

Immunohistochemical staining of immune and stellate cells was performed with paraffin-embedded sections of liver tissue. Leukocytes were stained with a monoclonal rat anti-mouse CD45 antibody (BD). Staining for T cells was performed with a monoclonal rabbit anti-mouse CD3 antibody (LabVision). Macrophages were analyzed by using a monoclonal rat anti-mouse F4/80 antibody (BMA Dianova). Analyzing the activation of stellate cells a monoclonal rabbit anti-mouse α-SMA antibody (Biomol/Epitomics) was used.

### Cell Proliferation Assay

The proliferation of hepatic stellate cell line GRX was determined with Cell Proliferation ELISA (Roche Applied Science). Cell Proliferation ELISA based on the measurement of BrdU incorporation during the DNA synthesis. The cells were starved in DMEM medium (PAA Laboratories) without FCS for 16 hours. Then, the cells were stimulated with 20 ng of recombinant CCL3 for 24 and 32 hours. After the stimulation, cells were labelled with BrdU for additional 2 hours. The cells were washed and fixed, and the amount of BrdU, which has been incorporated into the DNA, was detected by adding an anti-BrdU peroxidase antibody and TMB substrate solution. The substrate reaction was measured at a wavelength of 370 nm.

### Assessment of Cell Proliferation and Migration in a Scratch Assay

Additionally, hepatic stellate cell migration and proliferation were performed in a scratch assay. After the cells were confluent in 6-well plates, a straight scratch with a pipette tip (blue, Gilson) was created on the cell monolayer. The cells were stimulated for 24 and 32 hours with recombinant CCL3. To quantify cell migration and proliferation the width of the scratch was recorded and measured digitally (x100 magnification) before and after stimulation in order to calculate the percentage of regrowed area.

### Statistical Analysis

Data are depicted as mean ± SEM. Statistical significance was assessed by two-way analysis of variance followed by a Student’s *t* test, with Welch’s correction in case of unequal variances. *P* values under 0.05 were considered significant in all cases. Statistical analyses were performed using GraphPad Prism 5.

## Results

### CCL3^−/−^ Mice Displayed Ameliorated Liver Fibrosis

**Figure 1:**
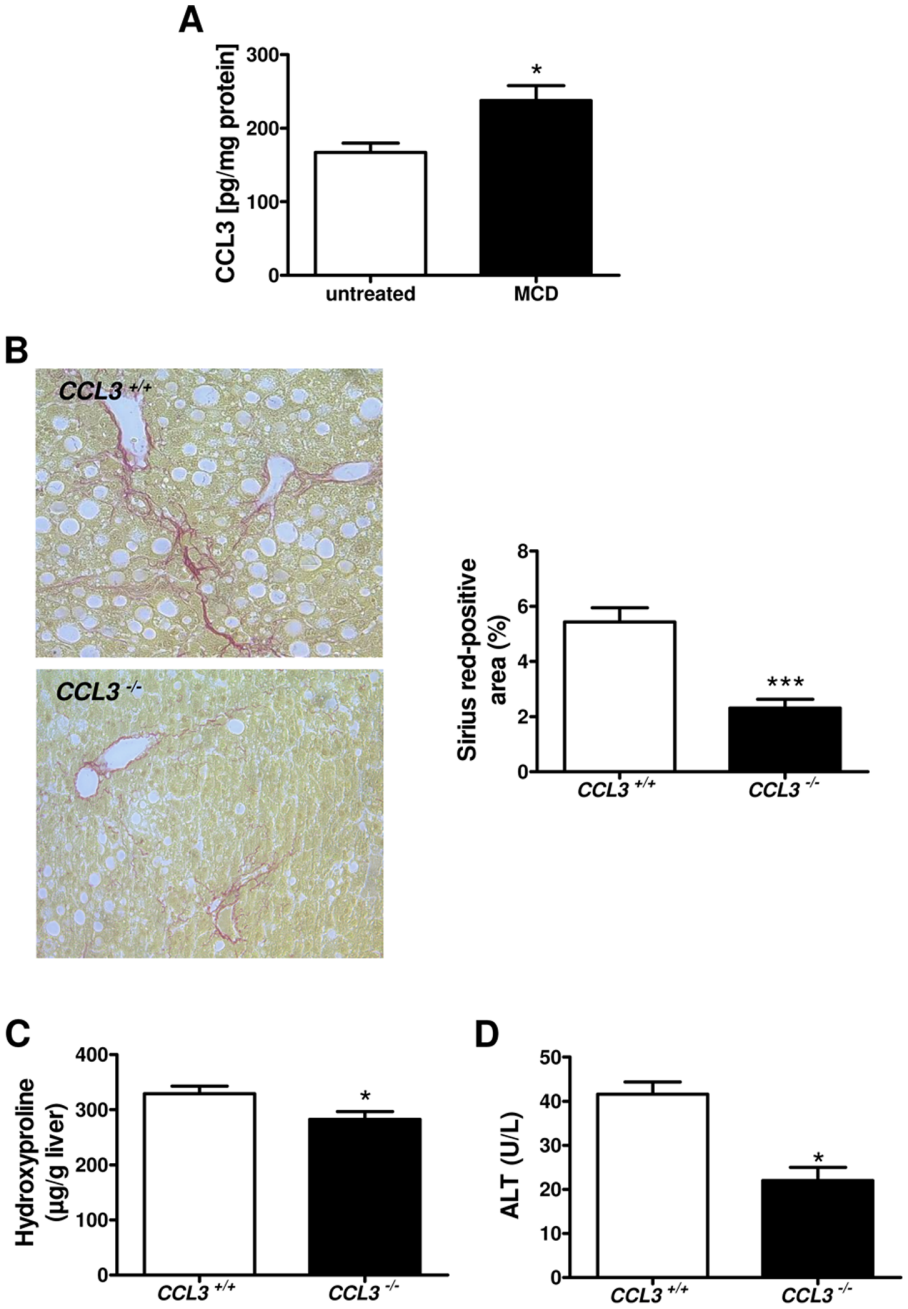
Liver fibrosis in *CCL3^−/−^* mice after eight weeks of MCD diet. CCL3 expression is increased in MCD-fed wild-type mice compared to untreated control (**A**). Representative Sirius red staining of *CCL3^−/−^* and wild-type (*CCL3^+/+^*) mice after MCD diet (x100 magnification). Quantitative analysis of the Sirius red-positive area showed a significantly decrease in *CCL3^−/−^* mice compared to wild-type mice (**B**). Hydroxyproline concentration in the liver (**C**) and serum level of ALT (**D**) were also significantly decreased in *CCL3^−/−^* mice. Data are expressed as means ± SEM of eight mice per group. *P<0.05, ***P<0.001.

First, we evaluated intrahepatic CCL3 levels before and after feeding a methionine- and choline-deficient (MCD) diet for eight weeks. We found that CCL3 protein expression is significantly increased after MCD diet when compared to untreated controls (Fig. 1A, *P*<0.05).

CCL3 is increased after MCD feeding; hence we tested if deletion of *CCL3* has an impact on liver fibrosis. *CCL3^−/−^* and wild-type (*CCL3^+/+^*) mice were fed a MCD diet for eight weeks. MCD-fed *CCL3^−/−^* animals showed significantly reduced liver fibrosis compared to *CCL3^+/+^* mice. This difference was evident after quantification of Sirius red stained liver tissues (Fig. 1B, *P*<0.001) and biochemical measurement of hepatic hydroxyproline contents (Fig. 1C, *P*<0.05). Serum levels of alanine aminotransferase (ALT) were also significantly lower in *CCL3^−/−^* animals (Figure 1D; *P*<0.05) after feeding with the MCD diet.

### Liver Injury in CCL3^−/−^ Mice is Associated with Altered Fibrosis and Lipogenesis Related Factors

**Figure 2:**
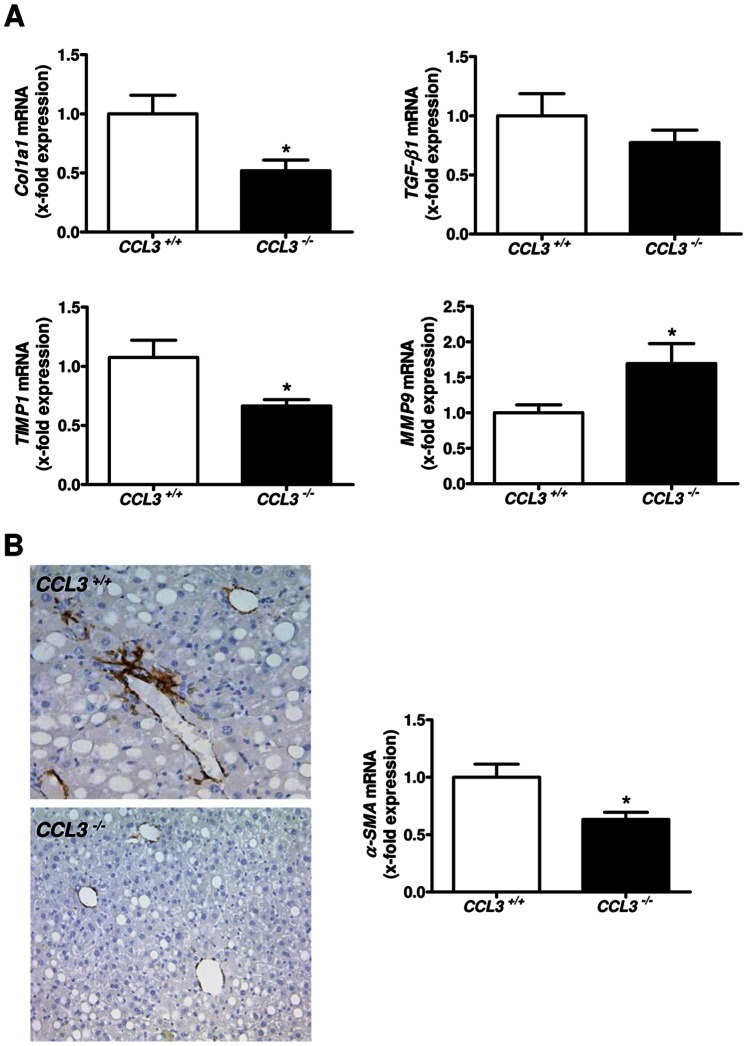
Fibrosis-related genes after eight weeks of MCD diet. MCD-fed *CCL3^−/−^* mice show a significantly altered expression of the fibrosis-related genes *Col1a1*, *TIMP1* and *MMP9* compared to wild-type mice. However, mRNA expression of *TGF-β1* does not vary in *CCL3^−/−^* mice (**A**). *α-SMA* expression is significantly decreased in *CCL3^−/−^* mice after MCD diet. Representative α-SMA staining is shown in the left panel (x100 magnification, **B**). Data are expressed as means ± SEM of eight mice per group. *P<0.05.

In a next step, we investigated the expression of fibrosis-related genes in the liver of MCD-fed *CCL3^−/−^* and *CCL3^+/+^* mice, which are involved in the deposition and remodelling of extracellular matrix. As depicted in figure 2A, reduced liver fibrosis in *CCL3^−/−^* mice was associated with altered expression of *Col1a1*, *TIMP1*, and *MMP9* (Fig. 2A, *P*<0.05). In contrast, mRNA expression of *TGF-β1* (Fig. 2A) and *MMP2* (data not shown) did not vary in *CCL3^−/−^* mice. Since these genes are also evident in the biology of hepatic stellate cells, we next assessed liver HSC activation after chronic liver injury. HSC activation was significantly reduced in *CCL3^−/−^* mice after MCD diet, as determined by immunohistochemical staining and quantitative real-time PCR analysis of α-SMA (Fig. 2B, *P*<0.05).

Moreover, *CCL3^−/−^* mice showed significantly reduced hepatic and serum triglyceride levels when compared to *CCL3^+/+^* mice (Figure S1A and B, *P*<0.05), suggesting that CCL3 might also operate in metabolic disease. These results were further confirmed by significantly reduced intraheptic mRNA expression of the lipogenic factors *SREBP1* and *Fas* (Figure S1C and D, *P*<0.05).

### CCL3^−/−^ Mice had Reduced Liver Fibrosis after CCl_4_ Challenge

**Figure 3:**
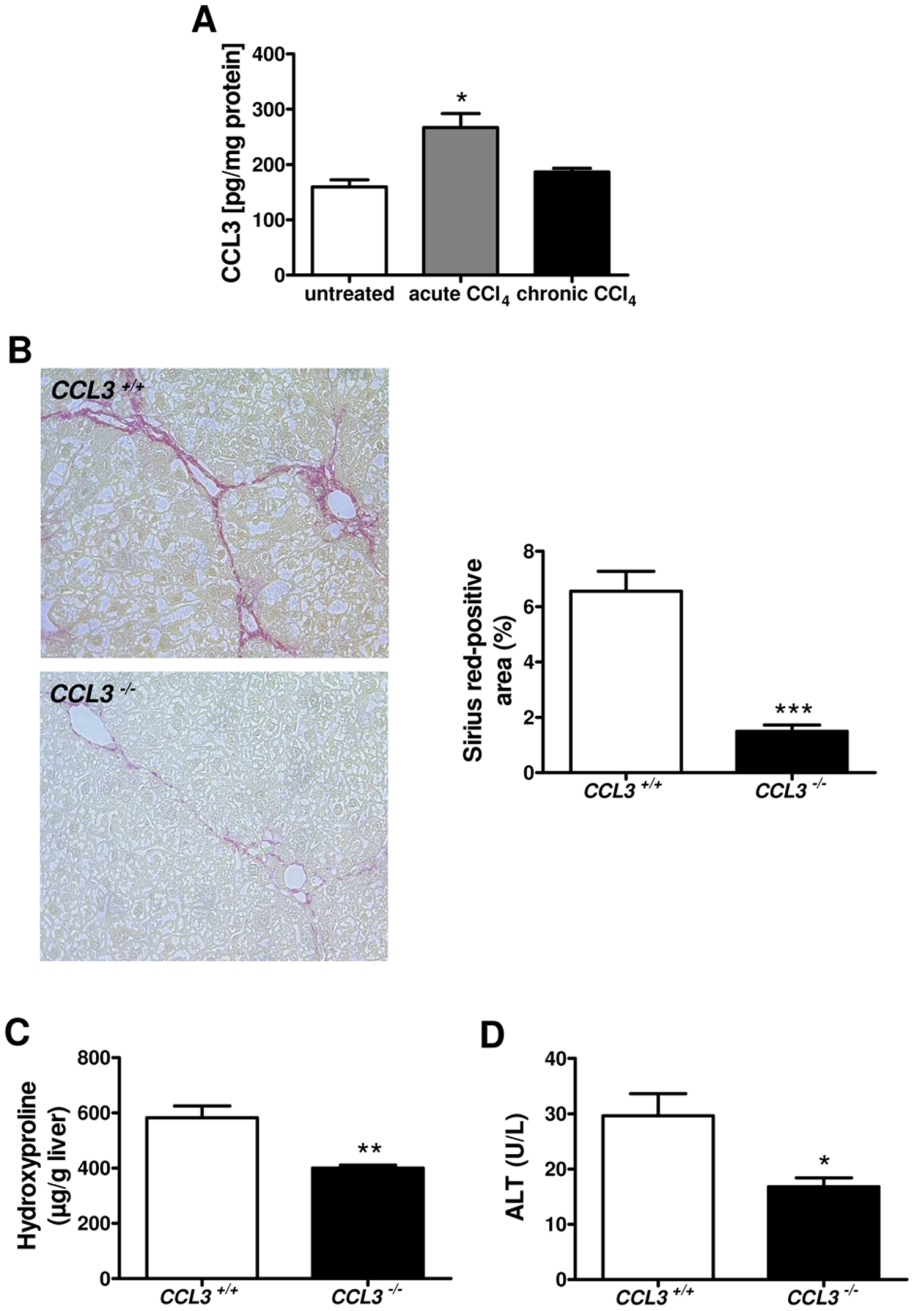
Reduced liver fibrosis in *CCL3^−/−^* mice after CCl_4_ challenge. Acute liver injury after CCl_4_ challenge is associated with augmented hepatic expression of CCL3, while CCL3 after chronic CCl_4_ treatment has a tendency toward higher expression (**A**). Sirius red staining after the CCl_4_ treatment (x100 magnification) shows reduced liver fibrosis in *CCL3* deficient mice (**B**). Hydroxyproline contents in the liver (**C**) and serum level of ALT (**D**) were significantly decreased in *CCL3^−/−^* mice. Data are expressed as means ± SEM of eight mice per group. *P<0.05, **P<0.01, ***P<0.001.

Next, we asked if the effect on dietary-induced liver damage also holds true in the CCl_4_ model of liver fibrosis. Intrahepatic CCL3 protein expression in untreated wild-type mice was compared to CCL3 protein level in wild-type mice after acute and chronic CCl_4_ treatment. As depicted in figure 3A, acute CCl_4_-mediated liver injury induced significantly higher CCL3 expression (*P*<0.05), while after chronic CCl_4_ treatment only a trend towards higher CCL3 expression was evident.

Along with the data in the MCD model histological analysis of Sirius red staining revealed reduced liver fibrosis in *CCL3* deficient mice after chronic CCl_4_ treatment (Fig. 3B, *P*<0.001). These results were further strengthened by markedly reduced concentrations of the collagen-specific amino acid hydroxyproline (Fig. 3C, *P*<0.01). Moreover, ALT values (Fig. 3D, *P*<0.05) and TUNEL^+^ cells within the liver (Figure S2A *P<0.05) were also reduced in *CCL3^−/−^* mice compared to wild-type control, suggesting a directly involvement of CCL3 in inflammatory hepatocyte damage.

### Liver Damage after Chronic CCl_4_ Treatment Led to Altered Expression of Fibrosis-Related Genes in CCL3^−/−^ Mice

**Figure 4:**
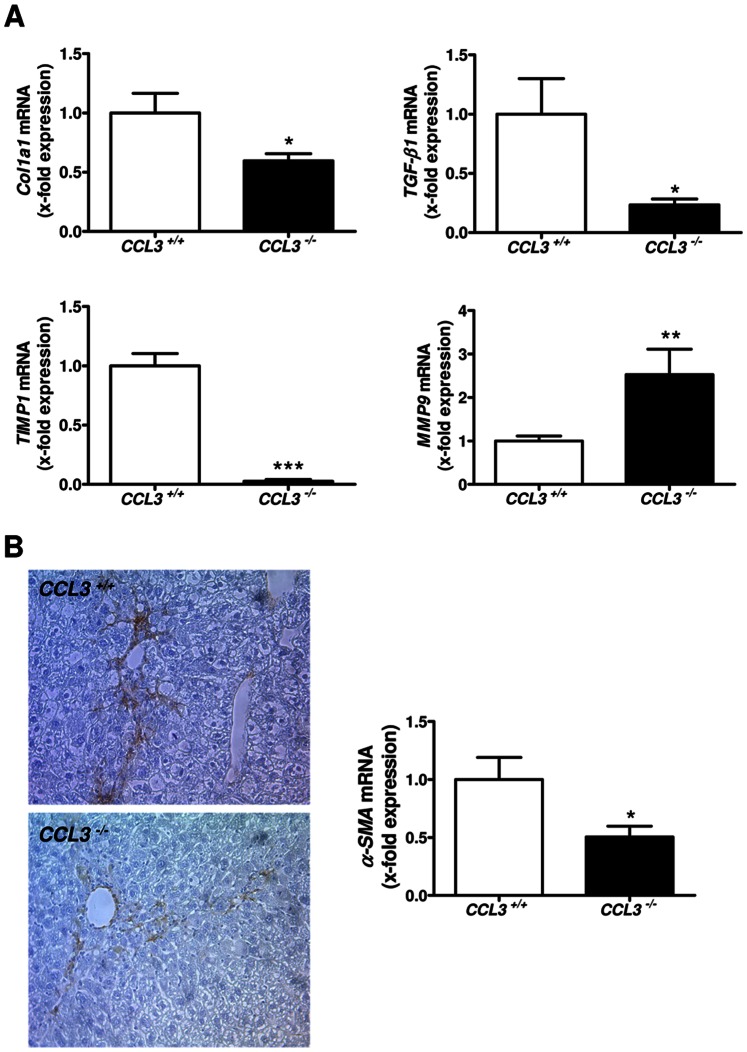
Altered expression of fibrosis-related genes in *CCL3^−/−^* mice. CCl_4_ treatment of *CCL3^−/−^* mice lead to significantly altered expression of the fibrosis-related genes *Col1a1, TGF-β1, TIMP1* and *MMP9* in *CCL3^−/−^* mice (**A**)**.** α-SMA expression is significantly decreased in *CCL3^−/−^* mice (x100 magnification, **B**). Data are expressed as means ± SEM of eight mice per group. *P<0.05, **P<0.01, ***P<0.001.

In liver fibrosis, one of the key events is HSC activation. To investigate this process, fibrosis-related genes, which are involved in HSC biology, were analyzed. mRNA expression of the fibrogenic genes *TGF-β1, Col1a1, TIMP1* and *MMP9* were significantly altered in *CCL3^−/−^* mice compared to their wild-type counterparts after chronic CCl_4_ treatment (Fig. 4A, *P*<0.05, *P*<0.001 and *P*<0.01, respectively), while *MMP2* levels were not significantly different between the two strains (data not shown). Alongside with data obtained in dietary-induced liver damage model, the level of α-SMA was also significantly reduced in *CCL3^−/−^* mice compared to controls (Fig. 4B). This difference was true after genetic (*P*<0.05) and immunohistochemical analyses of α-SMA. These findings suggest a model independent profibrotic mechanism of CCL3 during liver fibrosis.

### Inflammation-related Genes and the Number of T Cells are Reduced in CCL3^−/−^ Mice Treated with CCl_4_

**Figure 5:**
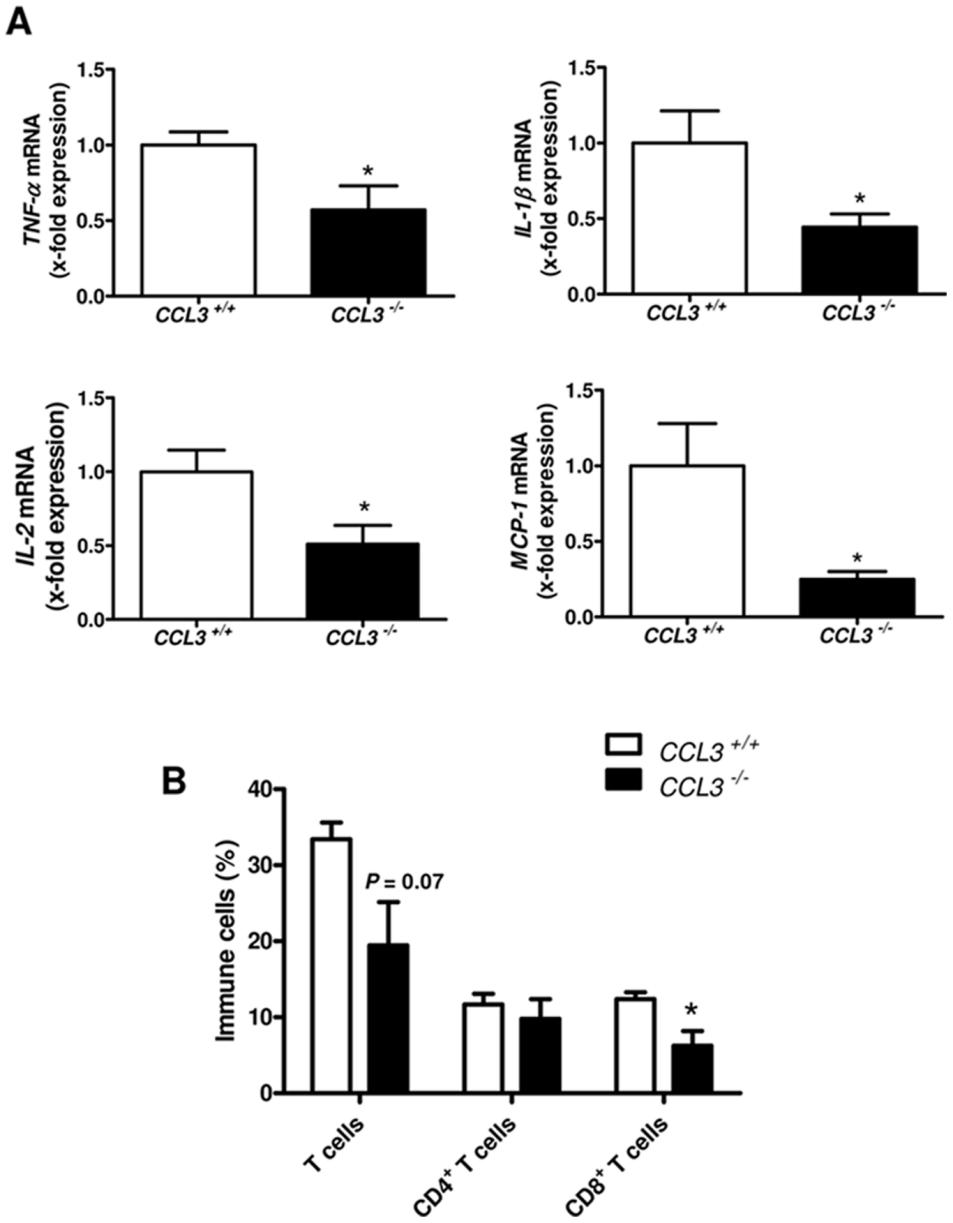
Inflammation is reduced in *CCL3^−/−^* mice treated with CCl_4_. *CCL3^−/−^* mice treated with CCl_4_ show altered expression of the inflammation-related genes *TNF-α, IL-1β, IL-2 and MCP-1* compared to wild-type mice (**A**). CD8^+^ T cell infiltration is significantly decreased in *CCL3^−/−^* mice (**B**). Data are expressed as means ± SEM of eight mice per group. *P<0.05.

**Figure 6:**
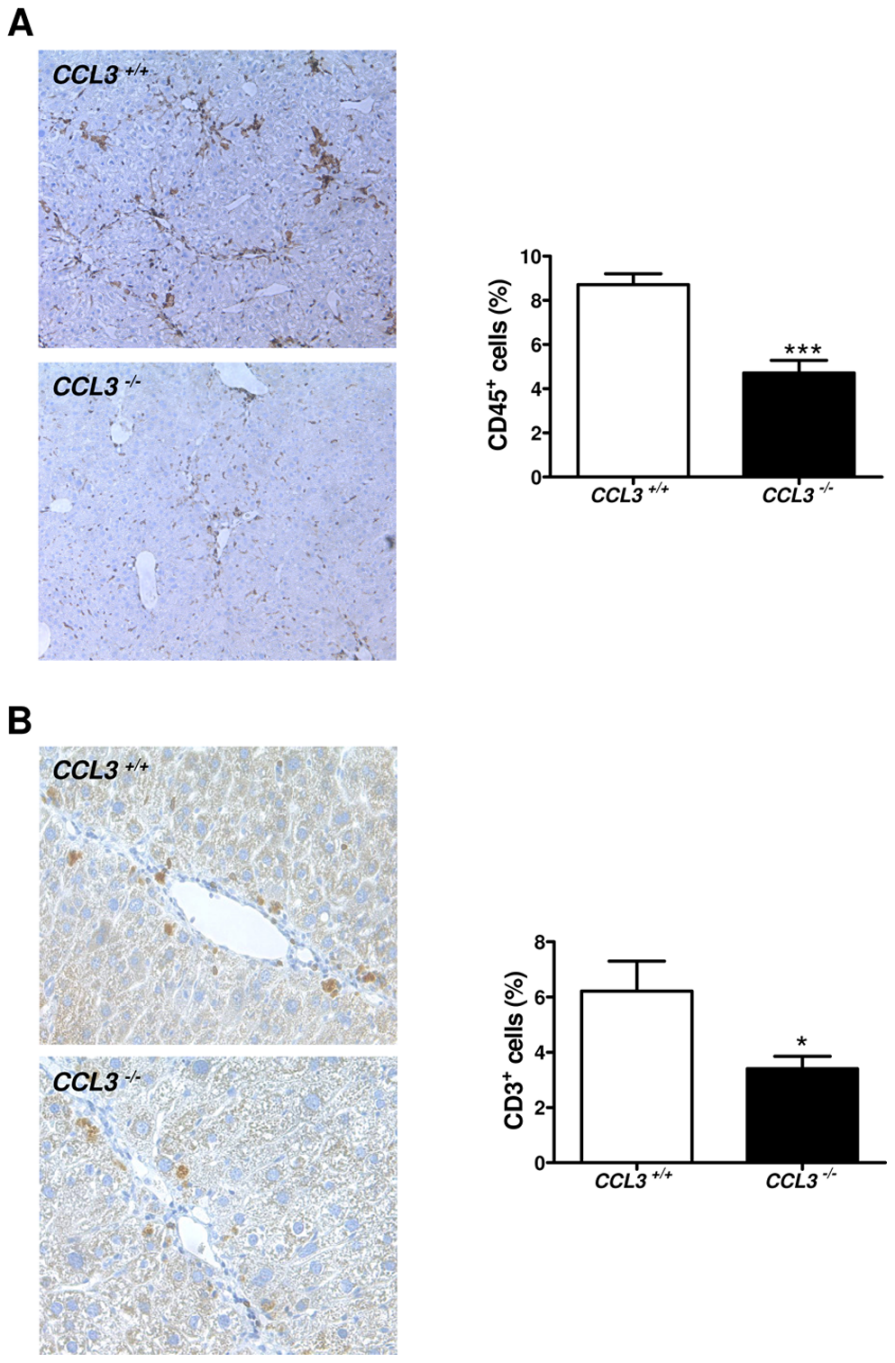
The lack of *CCL3* leads to reduced influx of CD45^+^ and CD3^+^ cells. CD45^+^ and CD3^+^cell infiltration is markedly reduced in *CCL3^−/−^* mice (x100 magnification, **A,** x200 magnification, **B**). Data are expressed as means ± SEM of eight mice per group. *P<0.05, ***P<0.001.

Immune cells contribute to liver fibrogenesis [24], [25]; we first investigated inflammation-related genes by quantitative RT-PCR analysis. Compared to WT mice, mRNA expression of *TNF-α*, *IL-1β*, *IL-2* and also *MCP-1 (monocyte chemoattractant protein-1, CCL2)* was markedly reduced in *CCL3* deficient mice (Fig. 5A, *P*<0.05). Consequently, we investigated immune cell infiltrating by FACS analysis and immunohistochemistry. FACS analysis revealed markedly reduced hepatic infiltration of T cells and, specifically of CD8^+^ T cells in *CCL3^−/−^* mice (Fig. 5B, *P* = 0.07 and *P* = 0.03, respectively), while the influx of NK and NKT cells were not strongly affected (data not shown). These results were further confirmed by quantification of absolute immune cell numbers within the liver (Figure S2B, *P* = 0.053 and P<0.05, respectively). By immunohistochemical quantification of representative liver sections, we found reduced numbers of CD45^+^ and CD3^+^ cells (Fig. 6A and B, *P*<0.001 and P<0.05, respectively) and also F4/80^+^ macrophages (Figure S3, *P*<0.05) within *CCL3* deficient livers treated with CCl_4_.

### CCL3 Accelerated the Proliferation of HSC *in vitro*

**Figure 7:**
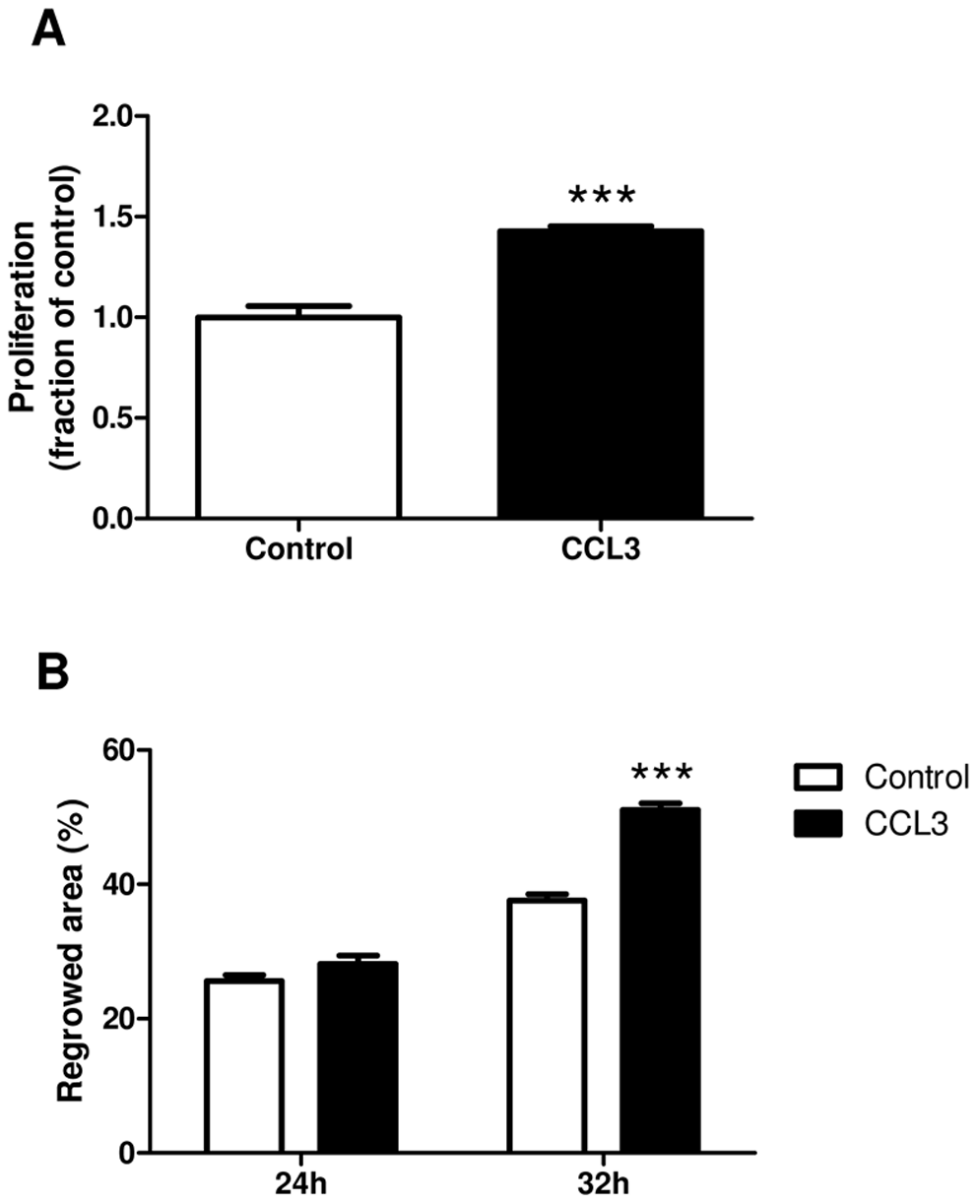
*In vitro* evidence for a role of CCL3 in liver fibrosis. Stimulation of hepatic stellate cell line (GRX) with recombinant CCL3 led to significantly increased proliferation compared to untreated cells (**A**). Stimulation of GRX cells with recombinant CCL3 showed significantly increased wound healing after 32 hours, while there is no difference after 24 hours between the groups (**B**). Data are expressed as means ± SEM of three independent experiments. ***P<0.001.

Finally, we investigated the direct effects of the chemokine on the immortalized hepatic stellate cell line GRX *in vitro*. As depicted in figure 7A, CCL3 significantly increased stellate cell proliferation (*P*<0.001), as determined by BrdU incorporation. Furthermore, CCL3 accelerated scratch closure triggered by enhanced proliferation and migration of stellate cells in a functional scratch assay (Fig. 7B and Figure S4). This effect was first observed 32 hours after incubation (*P*<0.001).

## Discussion

In our study, we demonstrate a pro-fibrotic effect of the CC chemokine CCL3 in two independent fibrosis models. Initially, we could show that CCL3 protein expression is significantly up-regulated in wild-type mice after methionine- and choline-deficient diet and acute CCl_4_ treatment, however, CCL3 protein expression was only moderate altered six weeks after CCl_4_ treatment. These observations are in accordance with data on pulmonary fibrosis [26] showing an increased intrapulmonary CCL3 level during the early inflammatory phase in response to thoracic irradiation and supported our assumption that liver resident and inflammatory cells-secreted CCL3 might facilitate immune cell recruitment into the liver, which in turn contributes to the degree of tissue injury [25].

This chemokine belongs to the intercrine beta (chemokine CC) family and binds to the receptors CCR1 and CCR5 [19], [27]. Mice lacking any of these receptors were less prone to hepatic fibrogenesis after chronic toxic injury or bile duct ligation [13]. These findings are in line with clinical studies showing an association between the *CCR5Δ32* polymorphism and the degree of the HCV-related hepatitis [28]. Furthermore, analysis of fibrogenesis in *CCR1* and *CCR5* chimeric mice revealed a divergent function of these chemokine receptors in the liver. Interestingly, CCR1 mediates its pro-fibrogenic effects predominantly through hematopoietic cells, whereas CCR5 mediates liver fibrosis mainly through resident liver cells, especially through hepatic stellate cells [13]. Their shared other ligand CCL5 is known to be involved in this process, as *CCL5* ^−/−^ mice also showed lower degree of experimental liver fibrosis associated with reduced stellate cell activation and immune cell infiltration [10]. Accordingly, systemic administration of Met-CCL5 or (44)AANA(47)-CCL5, a mutated CCL5 protein, led to a strong attenuation of experimental liver fibrosis *in vivo* [10], [29]. These findings, together with the knowledge that CCL3 is also a prominent ligand of the receptors CCR1 and CCR5, has brought us to further investigate the functional role of CCL3 during experimental liver damage.

Here, we could show that mice lacking *CCL3* showed significantly reduced MCD- and CCl_4_- induced liver fibrosis compared to *CCL3^+/+^* mice, as determined by quantification of Sirius red staining and biochemical measurement of hepatic hydroxyproline contents. These data were obtained in both fibrosis models *in vivo*, indicating that the functional mechanism of CCL3 is independent of the experimental model used. Notably, *CCL3* deficient mice also showed reduced ALT levels and TUNEL^+^ cells, assuming a direct involvement of CCL3 in inflammatory hepatocyte damage. The genetic deletion of *CCL3* indeed led to a reduced hepatic recruitment of T cells and macrophages after liver injury, confirming the hypothesis that reduced T cell and macrophage infiltrate might lead to less collateral damage in the liver, which in turn altered ALT levels [10]. These results are in line with earlier findings of the importance of CCL3 in models of pulmonary fibrosis [26] and acute liver injury [20].

In these mice, we also found less stellate cell activity, as determined by α-SMA mRNA and protein expression, an important marker for activated HSCs. These results suggest an interaction between immune and stellate cells which has been recently identified as an important event in liver fibrosis [4].

To further assess the functional aspect of CCL3 on hepatic stellate cells [30], we next stimulated these cells with recombinant CCL3 *in vitro*. Interestingly, CCL3 displayed strong proliferative effect on stellate cells, as determined by BrdU incorporation. Scratch assay analysis also showed significantly increased migration and proliferation of CCL3 treated cells compared to control, confirming these cells as resident target cell type of CCL3 [13]. However, with regard of fibrosis, additional studies will be needed to identify the temporal requirement of CCL3 for these actions.

Taken together, our findings present evidence that the CC chemokine CCL3 is a crucial mediator of experimental liver fibrosis. These preclinical results identify CCL3 as a potential molecular target for therapeutic strategies for chronic toxic and metabolic liver diseases.

## Supporting Information

Figure S1 **CCL3 is involved in metabolic disease.** *CCL3^−/−^* mice exhibits significantly lower hepatic and serum level of triglycerides compared to *CCL3^+/+^* mice after MCD diet (**A, B**). Altered triglyceride values were associated with reduced mRNA expression of *SREBP1* and *Fas* (**C, D**). Data are expressed as means ± SEM of eight mice per group. *P<0.05.
(TIF)

Figure S2 **Reduced liver injury and inflammation in** ***CCL3^−/−^*** **mice.** TUNEL^+^ cells (TUNEL staining, x100 magnification) within the liver were significantly reduced in *CCL3^−/−^* mice compared to *CCL3^+/+^* mice after six weeks of CCl_4_ treatment (**A**). Absolute CD45^+^, CD3^+^ and CD8^+^ cell numbers are markedly decreased in *CCL3^−/−^* mice (**B**). *P<0.05.
(TIF)

Figure S3 ***CCL3*** **deficiency results in reduced influx of macrophages.** F4/80^+^ cells (F4/80 staining, x100 magnification) within the liver were significantly decreased in *CCL3^−/−^* mice compared to *CCL3^+/+^* mice after six weeks of CCl_4_ treatment. *P<0.05.
(TIF)

Figure S4 **CCL3 accelerates proliferation and migration of stellate cells.** Representative pictures of scratch assay (x200 magnification). The pictures show the scratches after 0, 24 and 32 hours after the stimulation with 20 ng recombinant CCL3.
(TIF)
