# Supplementary figures and images for: The Chemokine CCL3 Promotes Experimental Liver Fibrosis in Mice
Source: PLoS One. 2013 Jun 17;8(6):e66106. doi: 10.1371/journal.pone.0066106 (PMC3684603; doi:10.1371/journal.pone.0066106)

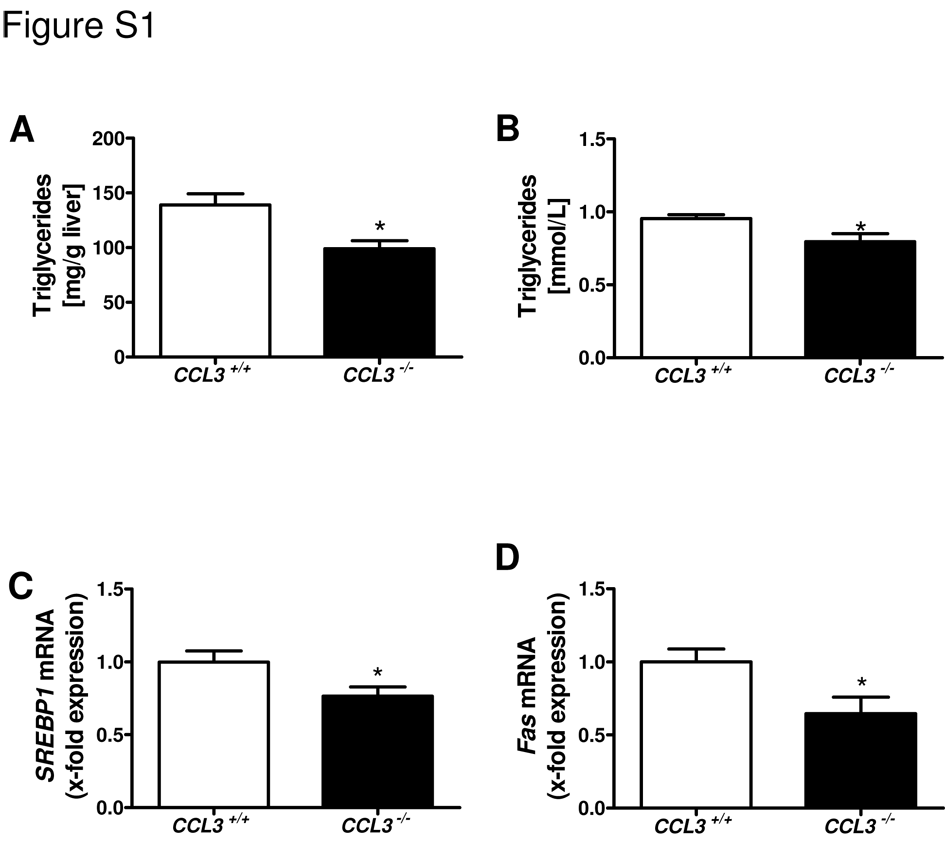

Supplement: Figure S1 — CCL3 is involved in metabolic disease. CCL3−/− mice exhibits significantly lower hepatic and serum level of triglycerides compared to CCL3+/+ mice after MCD diet (A, B). Altered triglyceride values were associated with reduced mRNA expression of SREBP1 and Fas (C, D). Data are expressed as means ± SEM of eight mice per group. *P<0.05. (TIF) [file pone.0066106.s001.tif]

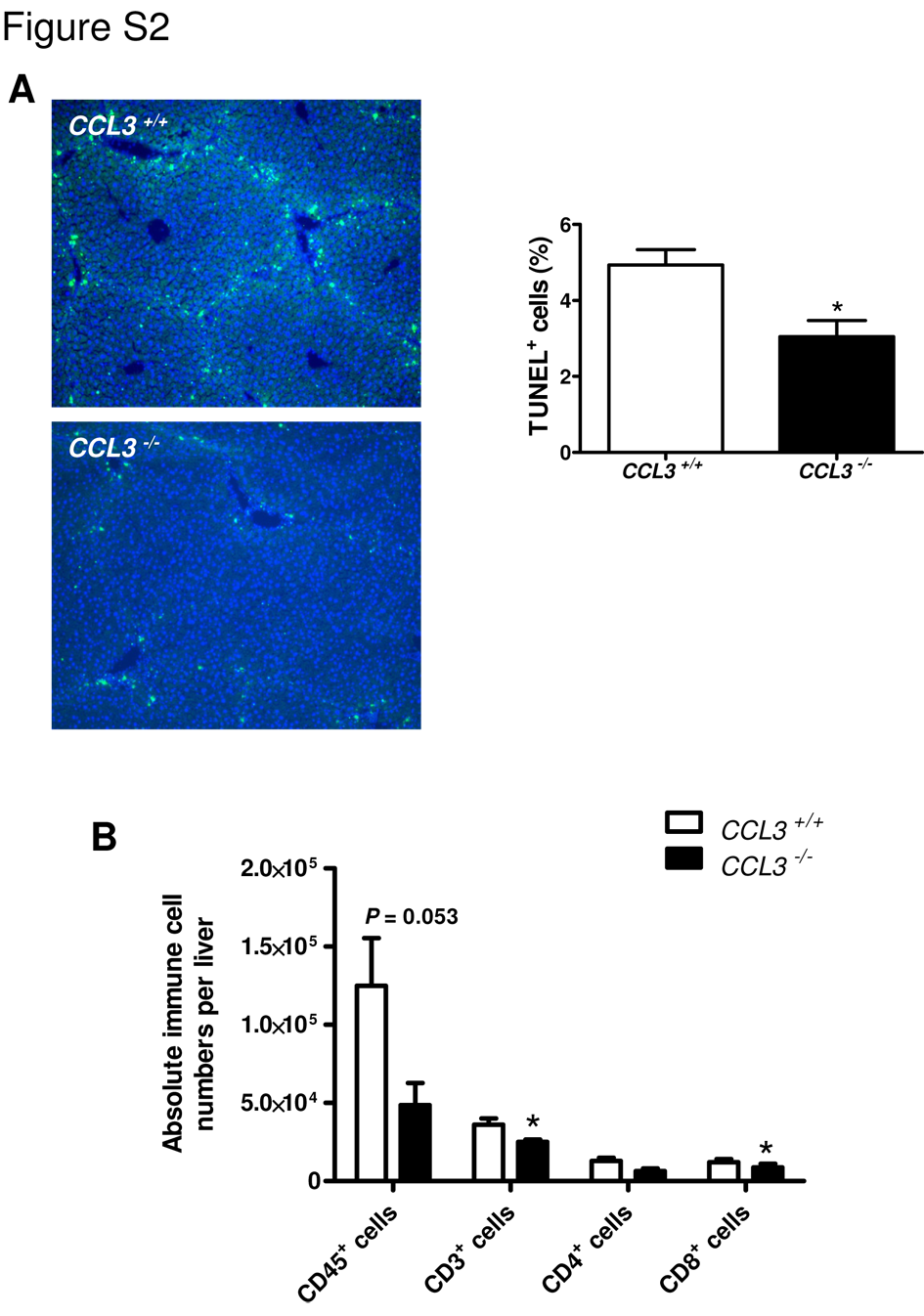

Supplement: Figure S2 — Reduced liver injury and inflammation in CCL3−/− mice. TUNEL+ cells (TUNEL staining, x100 magnification) within the liver were significantly reduced in CCL3−/− mice compared to CCL3+/+ mice after six weeks of CCl4 treatment (A). Absolute CD45+, CD3+ and CD8+ cell numbers are markedly decreased in CCL3−/− mice (B). *P<0.05. (TIF) [file pone.0066106.s002.tif]

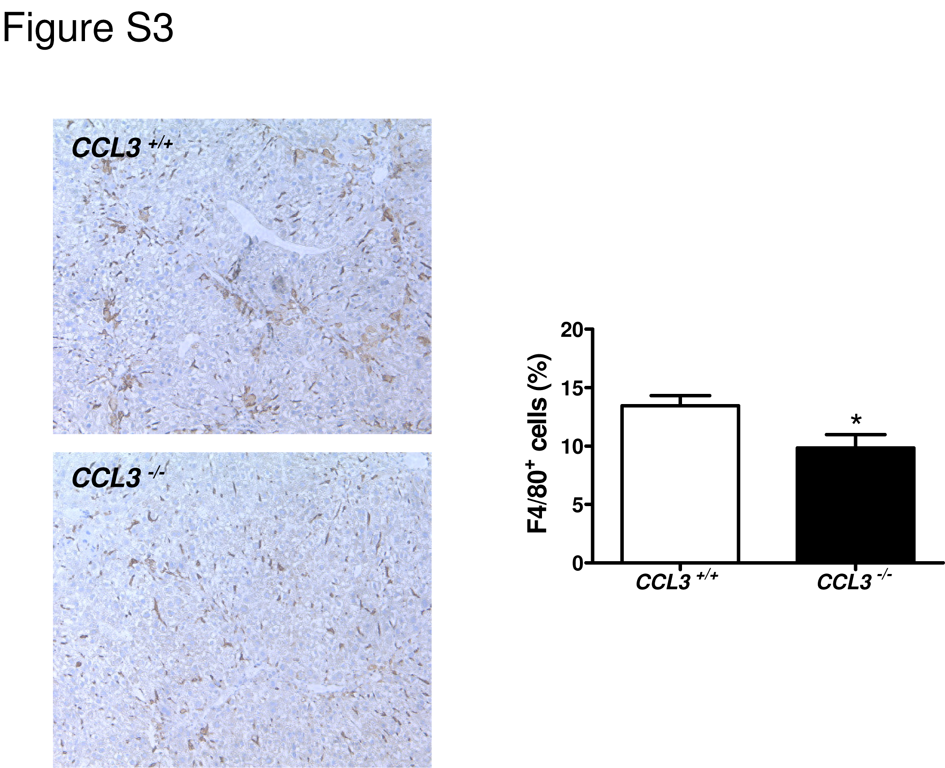

Supplement: Figure S3 — CCL3 deficiency results in reduced influx of macrophages. F4/80+ cells (F4/80 staining, x100 magnification) within the liver were significantly decreased in CCL3−/− mice compared to CCL3+/+ mice after six weeks of CCl4 treatment. *P<0.05. (TIF) [file pone.0066106.s003.tif]

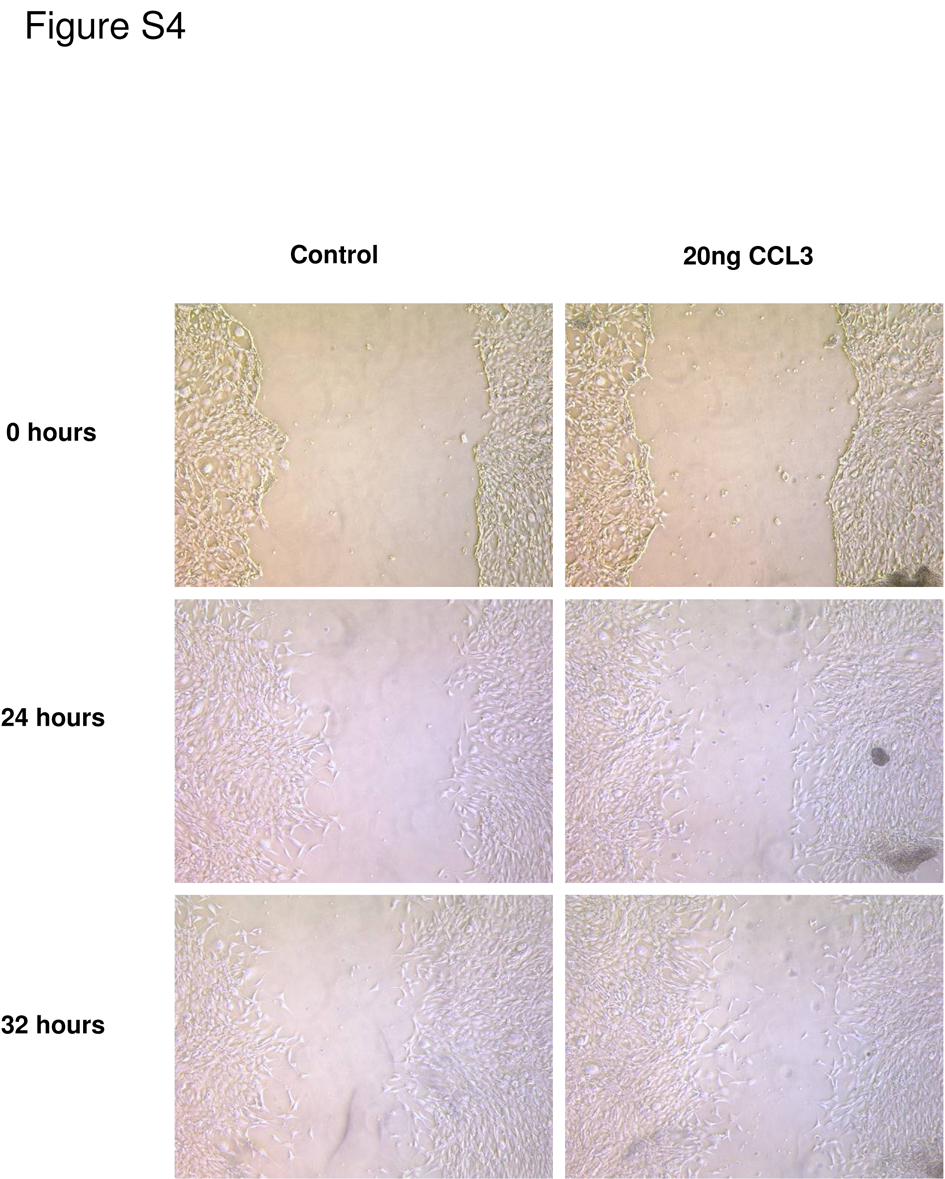

Supplement: Figure S4 — CCL3 accelerates proliferation and migration of stellate cells. Representative pictures of scratch assay (x200 magnification). The pictures show the scratches after 0, 24 and 32 hours after the stimulation with 20 ng recombinant CCL3. (TIF) [file pone.0066106.s004.tif]
